# The Epidemiologic and Pharmacodynamic Cutoff Values of Tilmicosin against *Haemophilus parasuis*

**DOI:** 10.3389/fmicb.2016.00385

**Published:** 2016-03-22

**Authors:** Peng Zhang, Haihong Hao, Jun Li, Ijaz Ahmad, Guyue Cheng, Dongmei Chen, Yanfei Tao, Lingli Huang, Yulian Wang, Menghong Dai, Zhenli Liu, Zonghui Yuan

**Affiliations:** ^1^National Reference Laboratory of Veterinary Drug Residues and MAO Key Laboratory for Detection of Veterinary Drug Residues, Huazhong Agricultural UniversityWuhan, China; ^2^MOA Laboratory for Risk Assessment of Quality and Safety of Livestock and Poultry Products, Huazhong Agricultural UniversityWuhan, China; ^3^Hubei Collaborative Innovation Center for Animal Nutrition and Feed Safety, Huazhong Agricultural UniversityWuhan, China

**Keywords:** epidemiologic cutoff value, pharmacodynamic cutoff, bronchoaveolar lavage, *H. parasuis*, tilmicosin

## Abstract

The aim of this study was to establish antimicrobial susceptibility breakpoints for tilmicosin against *Haemophilus parasuis*, which is an important pathogen of respiratory tract infections. The minimum inhibitory concentrations (MICs) of 103 *H. parasuis* isolates were determined by the agar dilution method. The wild type (WT) distribution and epidemiologic cutoff value (ECV) were evaluated by statistical analysis. The new bronchoaveolar lavage was used to establish intrapulmonary pharmacokinetic (PK) model in swine. The pharmacokinetic (PK) parameters of tilmicosin, both in pulmonary epithelial lining fluid (PELF) and in plasma, were determined using high performance liquid chromatography method and WinNonlin software. The pharmacodynamic cutoff (CO_PD_) was calculated using Monte Carlo simulation. Our results showed that 100% of WT isolates were covered when the ECV was set at 16 μg/mL. The tilmicosin had concentration-dependent activity against *H. parasuis*. The PK data indicated that tilmicosin concentrations in PELF was rapidly increased to high levels at 4 h and kept stable until 48 h after drug administration, while the tilmicosin concentration in plasma reached maximum levels at 4 h and continued to decrease during 4–72 h. Using Monte Carlo simulation, CO_PD_ was defined as 1 μg/mL. Conclusively, the ECV and CO_PD_ of tilmicosin against *H. parasuis* were established for the first time based on the MIC distribution and PK-PD analysis in the target tissue, respectively. These values are of great importance for detection of tilmicosin-resistant *H. parasuis* and for effective treatment of clinical intrapulmonary infection caused by *H. parasuis*.

## Introduction

*Haemophilus parasuis*, a Gram-negative bacterium of the *Pasteurella* group of organisms, is a respiratory pathogens of pigs (Fu et al., 2013; Luan et al., 2013). It is mainly responsible for Glässer’s disease, which is characterized by polyserositis, arthritis, and meningitis (Guo et al., 2011; Mullins et al., 2013; Moleres et al., 2015) and has caused large economic losses in the worldwide pig industry in recent years (Vilalta et al., 2012; Zehr et al., 2012).

Tilmicosin is a macrolide antibiotic for veterinary use, which is semi-synthesized by tylosin hydrolysate. Similar to other macrolides, this drug has a long half-life and maintains high concentrations both in lung and milk. Tilmicosin has high-activity against respiratory pathogens such as *Pasteurella multocida*, *Actinobacillus pleuropneumoniae*, and *H. parasuis* (DeRosa et al., 2000). In addition, the antimicrobial activity of tilmicosin is much better than that of tylosin (Ayling et al., 2000). Therefore, tilmicosin has broad clinical application for treatment of respiratory diseases in animals (Frank et al., 2000; Naccari et al., 2001; Fittipaldi et al., 2005).

Establishing the antimicrobial susceptibility breakpoint sets the basis for antimicrobial susceptibility testing and antimicrobial resistance surveillance. Although breakpoints of tilmicosin against *P. multocida* and *A. pleuropneumoniae* are available in Clinical and Laboratory Standards Institute (CLSI) standards, a breakpoint of tilmicosin against *H. parasuis* has not yet been established.

An appropriate antimicrobial susceptibility breakpoint is established by the interpretation of four main types of data (Turnidge and Paterson, 2007): (i) MIC distributions; (ii) resistance markers; (iii) PK/PD data of the target subjects; (iv) clinical data of wild type (WT) isolates which has no reported detectable resistance mechanisms (Turnidge et al., 2006). The statistical analysis are used in setting breakpoints (Turnidge et al., 2006; Kronvall, 2010; Meletiadis et al., 2012). Without clinical data, an epidemiologic cutoff value (ECV) could be used to discriminate WT strains from isolates with resistance mechanisms (Espinel-Ingroff et al., 2010). However, a susceptibility breakpoint solely based on ECV could not predict the clinical outcome. In the absence of a clinical cutoff, the Pharmacokinetic/Pharmacodynamic cutoff (CO_PD_) is associated with clinical efficacy, as both PK/PD data and WT distribution are included for setting CO_PD_.

The plasma concentration of the drug is responsible for pharmacological effect. Several studies have shown that the drug concentrations in the target sites were directly correlated with clinical efficacy (Barbour et al., 2010). In addition, bronchoaveolar lavage (BAL) has frequently been used to establish an intrapulmonary PK model, especially for macrolides (Conte et al., 2000b, 2004). In the case of animals, the problem of long sampling intervals makes plasma concentrations incomparable with pulmonary epithelial lining fluid (PELF) concentrations (Villarino et al., 2013).

The purpose of this study was to establish the ECV and CO_PD_ of tilmicosin against *H. parasuis*, in addition to an intrapulmonary PK model of tilmicosin in pigs.

## Materials and Methods

### Organisms

From March to May 2014, a total of 103 *H. parasuis* strains were donated by State Key Laboratory of Agricultural Microbiology at Huazhong Agricultural University and National Reference Laboratory of Veterinary Drug Residues at South China Agricultural University. These strains were isolated from the lungs of swine in 10 provinces of China. All of the bacterial isolates were confirmed by polymerase chain reaction (PCR). Prior to testing, each isolate was subcultured at least twice on tryptic soy agar (TSA) containing 5% fetal calf serum (FCS) and 10 μg/mL nicotinamide adenine dinucleotide (NAD) to ensure viability and purity.

### Antimicrobial Susceptibility Determination

Susceptibility testing was performed by the agar dilution method according to the CLSI M07-A9 standard with some modification based on the characteristics of *H. parasuis*. A 2 μL *H. parasuis* suspension (10^8^ CFU/mL) was inoculated onto TSA-FCS-NAD agar plates containing twofold dilutions (0.015~32 μg/mL) of tilmicosin (Dr. Ehrenstorfer Standards, Augsburg, Germany). Plates were incubated at 37°C in an atmosphere containing 5% CO_2_ for 36 h. *Enterococcus faecalis* (ATCC 29212) was used as the quality control (QC) strain to ensure the credibility of MICs tested.

### Definition of Wild Type Cutoff (CO_WT_) or Epidemiologic Cutoff (ECV)

A microorganism is defined as WT for a species by the absence of resistance mechanisms to target drug (Turnidge et al., 2006; Turnidge and Paterson, 2007). The ECV, or CO_WT_ is used to separate bacterial populations on the basis of MIC distributions (Turnidge and Paterson, 2007; Espinel-Ingroff et al., 2010; Canton et al., 2012). Ideally, at least 95% of WT isolates should be encompassed into the ECV (Pfaller et al., 2009, 2010, 2011). The ECV was calculated following the method described by Turnidge (Turnidge et al., 2006). Briefly, Normality testing of the WT distribution was conducted with Sigmastat software v.3.5, non-linear regression was used to fit log_2_-transformed MICs with Graphpad Prism v.5.01, NORMINV and NORDIST functions were employed to set the WT distribution cutoffs.

### Determination of Possible Resistance Mechanism

The molecular mechanism involved in macrolide resistance in *H. parasuis* is unclear, however, mutations in the 23S rRNA that is commonly associated with macrolide-resistance may occur in *H. parasuis*. Therefore, PCR amplification (Hao et al., 2013) with the primers 23S-F (5′-ACGGTCCTAAGGTAGCGAAAT-3′) and 23S-R (5′-CATCAAATGTTAAAGGGTGGTA-3′) was used to screen mutations in the 23S rRNA gene in *H. parasuis.*

### *In vitro* Time Killing Test

According to the determined MIC of *H. parasuis* SH0165, agar plates were prepared with concentrations of tilmicosin (Dr. Ehrenstorfer standards, Augsburg, Germany)) ranging from 1/4 to 32 MIC. A total of 0.1 mL of inoculum was plated and bacterial counts were performed at 0, 1, 2, 4, 8, 12, and 24 h.

### Animals

Fourteen 10-weeks-old healthy crossbred (Duroc × Large white × Landrace) pigs weighing 28–33 kg were purchased from Huazhong Agricultural University pig breeding farm. Prior to experiments, pigs were raised 7 days to acclimate. Two pigs were used for establishment of high performance liquid chromatography (HPLC) method, another 12 pigs were used for pharmacokinetics (PK) study.

All the animal experiments were approved by the Animal Ethics Committee of Huazhong Agricultural University (hzauch 2014-003) and the Animal Care Center, Hubei Science and Technology Agency in China (SYXK 2013–0044). All efforts were used to reduce the pain and adverse effect of the animals.

### PK Study Design

The 12 pigs were randomly divided into two groups. Tilmicosin was orally administrated to six pigs in each group at a single-dose of 40 mg/kg/bw. This dose was designed based on the clinical outcome of 400 mg/kg feed (about 40 mg/kg/bw) for treatment of *H. parasuis* in pigs (MacInnes et al., 2003). In Group A, blood (2 mL) samples from animals were obtained at 0, 0.5, 0.75, 1, 2, 3, 4, 5, 6, 8, 12, 24, 36, 48, 72, and 96 h after administration. In Group B, PELF samples were collected at 0, 0.5, 2, 4, 6, 8, 12, 24, 36, 48, 72, and 96 h post-drug administration.

### Anesthesia Scheme and Bronchoaveolar Lavage (BAL)

Atropine (0.05 mg/kg), ketamine (5 mg/kg), and propofol (3 mg/kg) were given intramuscularly and intravenously 30 min before drug administration.

Standardized BAL was performed as previously described (Yamazaki et al., 2003; Choi et al., 2012; Lee et al., 2015), with an electronic fiberoptic bronchoscope (Kangmei GU-180VET) inserted in the right middle lung lobe. Then, 50 mL of normal saline was instilled into the lobe, and was aspirated into a 50 mL centrifugal tube after 20 s.

### Specimen Handling

Plasma was separated from blood by centrifugation at 3000 × *g* for 10 min and was kept at -70°C until assay. The PELF was centrifuged at 400 × *g* for 10 min and stored at -70°C until analysis.

### Tilmicosin Assay Using High Performance Liquid Chromatography (HPLC)

Quantitation analysis of tilmicosin in PELF and plasma were conducted using HPLC. A C_18_ reverse-phase column (250 mm × 4.6 mm i.d., 5 μm; Agilent) was used to perform HPLC at 30°C. The detection wavelength was 285 nm. The mobile phase consisted of 5 mM ammonium formate added with 0.1% formic acid (phase A) and acetonitrile (phase B; 73:27, v/v).

Plasma (0.5 mL) was extracted with dichloromethane (2.5 mL) twice. After centrifugation, supernatant was evaporated and resuspended in the mobile phase (0.5 mL). PELF (0.5 mL) was extracted with acetonitrile (2 mL), and then centrifuged, evaporated and resuspended in a same manner.

The limit of determination (LOD) was 0.02 μg/mL and the limit of quantification (LOQ) was 0.05 μg/mL both in plasma and PELF. Standard curves were linear from 0.05 to 10 μg/mL both in plasma (*R*^2^ = 0.9998) and PELF (*R*^2^ = 0.9999). The inter-day variation for determination in plasma ranged from 0.78 to 1.14% and PELF 0.14 to 0.78%, respectively. The recovery of tilmicosin in plasma ranged from 93.67 ± 1.06% to 97.16 ± 1.00% and in PELF from 98.49 ± 0.77 to 99.44 ± 014%.

### Quantitation of PELF Volume

The urea dilution method was used to determine the volume of PELF as described previously (Conte et al., 2000a; Kiem and Schentag, 2007). The concentration of urea in plasma and PELF were determined by the urease-glutamate dehydrogenase enzymatic method with an automatic biochemical analyzer (SYNCHRON CX4 PRO; Beckman) at the National Reference Laboratory of Veterinary Drug Residues (Wuhan, China).

### Pharmacokinetics Analysis

Statistical analysis was conducted by using WinNonlin v. 5.2.1. Plasma concentration data was analyzed with a two-compartment model and PELF non-compartment model according to the characteristics of concentration-time data.

### Monte Carlo Simulation and CO_PD_

Crystal Ball v7.2.2 was used to perform Monte Carlo simulation. The mean value and standard deviation of AUC_24_ of PELF were embedded in the function. The distribution of pharmacokinetic parameter AUC_24_ was assumed to be log-normal. A total of 10000 subjects were simulated.

The PK/PD target was evaluated (Andes et al., 2004). Conservative value (AUC/MIC = 30) was selected to calculate the probability of target attainment (PTA). CO_PD_ was defined as the MIC at which the PTA was ≥90%.

## Results

### Wild-Type (WT) MIC Distribution

All presumptive *H. parasuis* isolates were confirmed by PCR-based method. The MIC for *Enterococcus faecalis* (ATCC 29212) was 8 μg/mL, which was within the acceptable QC range according to CLSI document M31-A3.

**FIGURE 1:**
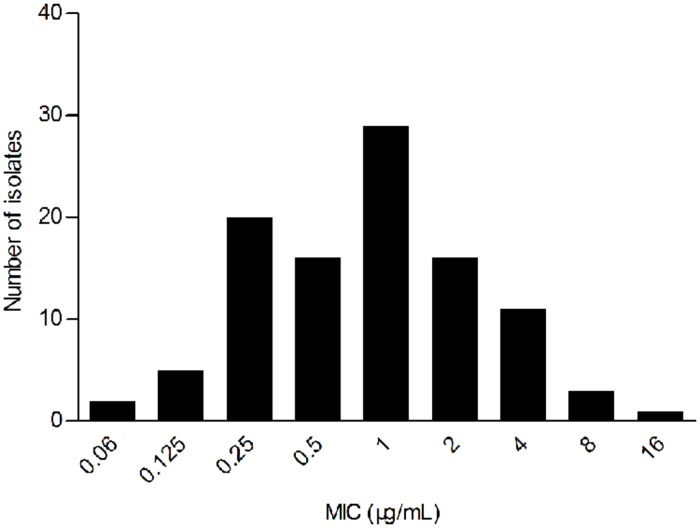
**Primary MIC distribution of tilmicosin against 103 *Haemophilus parasuis* isolates**.

The WT MIC distribution for tilmicoin against *H. parasuis* is shown in **Figure 1**. The tilmicosin MIC ranged from 0.06 to 16 μg/mL. The distribution of each MIC (0.06, 0.125,0.25, 0.5, 1, 2, 4, 8, and 16 μg/mL) among tested isolates was as follows: 0.06 μg/mL (1.94%), 0.12 μg/mL (4.85%), 0.25 μg/mL (19.42%), 0.5 μg/mL (15.53%), 1 μg/mL (28.16%), 2 μg/mL (15.53%), 4 μg/mL (10.68%), 8 μg/mL (2.91%), and 16 μg/mL (0.97%). The MIC_50_ and MIC_90_ were 1 and 4 μg/mL, respectively.

### The Epidemiologic Cutoff Value (ECV)

**FIGURE 2:**
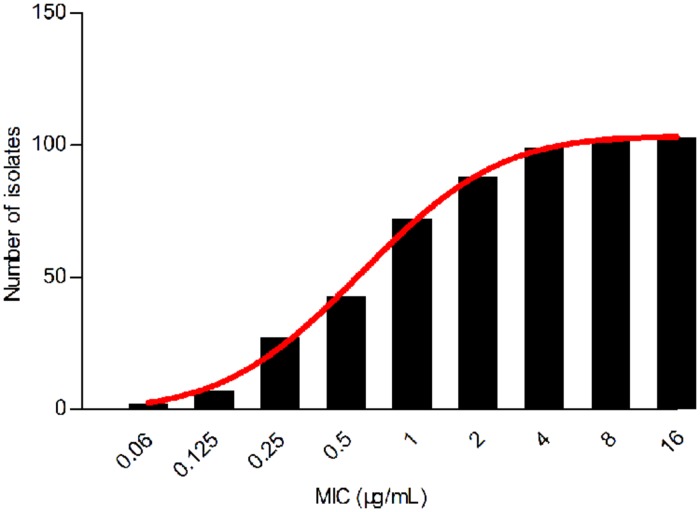
**Cumulative MIC distribution of tilmicosin against *H. parasuis* after non-linear regression**.

Despite a lower prevalence at the MIC of 0.5 μg/mL, cumulative counts of MIC data were found to match a good-shape normal distribution by use of normality test (*P* = 0.200) as shown in **Figure 2**. The optimum MIC range (0.015–16 μg/mL) was obtained using non-linear regression (**Table 1**). In addition, this range was further corrected to 0.06–16 μg/mL by employing the NORMINV function. The probabilites of an isolate MIC value higher than the high cutoff (0.18%) and lower than the low cutoff (0.07%) were estimated using NORMDIST function. As the result, the ECV was defined as 16 μg/mL, which encompassed 100% of the WT isolates (**Table 1**).

**Table 1:** Optimum non-linear least squares regression fitting of pooled MICs (μg/mL) for tilmicosin and *Haemophilus parasuis.*

Subset fitted	Number of isolates						Mean MIC (log_2_)				Standard deviation (log_2_)			
	True	Est.	Diff.	ASE	Est./ASE	95% CI^b^	Est.	ASE	Est./ASE	95% CI^a^	Est.	ASE	Est./ASE	95% CI^b^
≦1	72	120	48	59.930	2.0	-138, 378	-0.443	1.182	-0.3	-5.530, 4.645	1.802	0.630	2.9	-0.910, 4.514
≦2	88	102	14	10.180	10.0	69, 134	-0.810	0.268	-3.0	-1.661, 0.042	1.615	0.236	6.8	0.864, 2.366
≦4	99	103	4	4.352	23.7	91, 116	-0.765	0.134	-5.7	-1.136,-0.393	1.648	0.147	11.2	1.239, 2.057
≦8	102	103	1	2.334	44.1	92, 109	-0.773	0.086	-9.0	-0.995,-0.551	1.640	0.107	15.3	1.364, 1.916
≦16^b^	103	103	0	1.565	65.8	99, 107	-0.774	0.068	-11.4	-0.940,-0.608	1.639	0.088	18.6	1.423, 1.855

*Est., non-linear regression estimate of value; Diff., estimate of N minus true N; ASE, asymptotic standard error; Est./ASE, estimate divided by asymptotic standard error. ^a^95% CI of estimate of value. ^b^This subset gave the smallest difference between the estimate and true number of isolates in the subset.*

After PCR amplification and DNA sequencing, no 23S rRNA gene mutations associated with macrolide resistance were found among all of the tested *H. parasuis* isolates. Therefore, all isolates were assumed to be WT strains.

### *In vitro* Time Kill Curve

**FIGURE 3:**
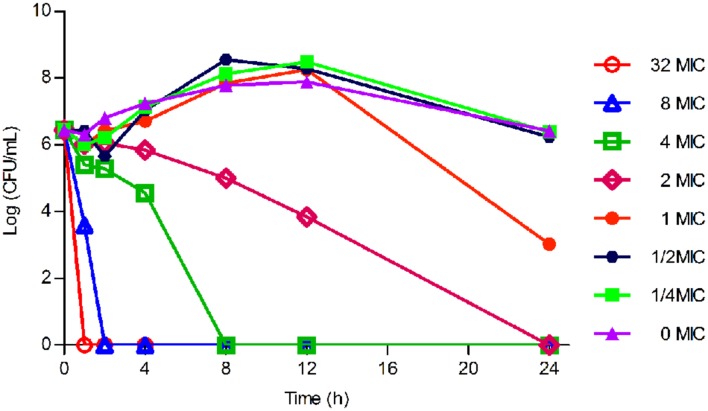
**The in *vitro* time killing curve of tilmicosin against *H. parasuis***. Fixed concentration (10^6^CFU/mL) of *H. parasuis* was incubated with different concentration of tilmicosin (1/4 MIC, ½ MIC, ……, 32 MIC). The X-axis was the different time point and the Y-axis was log_10_ transformed number of *H. parasuis*.

As displayed in **Figure 3**, the lower concentrations (≤MIC) of tilmicosin exhibited similar antimicrobial activity to *H. parasuis*. However, when tilmicosin concentrations were higher than MIC, the bacteriostatic efficiency gradually strengthened due to increased drug concentration. Therefore, the *in vitro* time kill curve showed that activity of tilmicosin against *H. parasuis* was concentration-dependent.

### Pharmacokinetic Characteristics of Tilmicosin in Plasma

**FIGURE 4:**
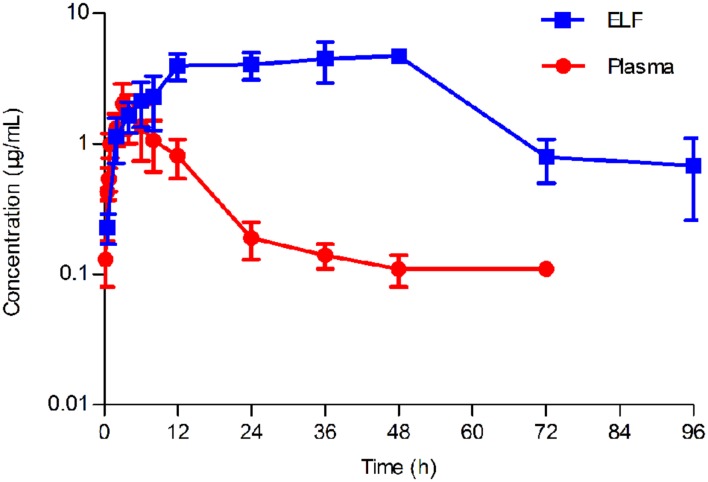
**Concentration-time curve of tilmicosin in plasma and in epithelial lining fluid (ELF) after oral administration at a single dose of 40 mg/kg to pigs (*n* = 6).** The concentration of tilmicosin in plasma was determined at 0, 0.5, 0.75, 1, 2, 3, 4, 5, 6, 8, 12, 24, 36, 48, 72, and 96 h after drug administration. The concentration of tilmicosin in ELF was detected at 0, 0.5, 2, 4, 6, 8, 12, 24, 36, 48, 72, and 96 h post-drug administration.

No serious adverse influences were observed after oral administration of tilmicosin. The concentration of tilmicosin in plasma was reduced below the LOQ after 72 h. A two-compartment model was used for model fitting from 0 to 72 h. The concentration and time profiles are illustrated in **Figure 4**. Pharmacokinetic parameters of tilmicosin in plasma were calculated by model analysis conducted by WinNonlin v. 5.2.1. As shown in **Table 2**, the time to reach to maximum concentration (Tmax), the peak drug concentration (*C*max), and the area under the curve at 24 h (AUC_24_) were 3.52 ± 0.34 h, 1.57 ± 0.46 μg/mL, and 20.13 ± 5.26 μg. h/mL, respectively. The mean residence time (MRT) was 16.45 ± 1.67 h. The intercept for the distribution phase (A) and for the elimination phase (B) were 2.67 ± 0.99 μg/mL and 0.09 ± 0.01 μg/mL. The distribution rate constant (α) and elimination rate constant (β) were 0.11 ± 0.01 L/h and 0.002 ± 0.001 L/h.

**Table 2:** Pharmacokinetic parameters in plasma and ELF after oral administration of tilmicosin at a single dose of 40 mg/kg.bw.

Parameters	Mean ± SD values	
	Plasma	ELF
AUC_24_ (μg.h/mL)	20.13 ± 5.26	74.41 ± 17.98
T_max_ (h)	3.52 ± 0.34	40.80 ± 6.57
C_max_ (μg/mL)	1.57 ± 0.46	5.36 ± 0.74
A (μg/mL)	2.67 ± 0.99	
B (μg/mL)	0.09 ± 0.01	
α (L/h)	0.11 ± 0.01	
β (L/h)	0.002 ± 0.001	
MRT (h)	16.45 ± 1.67	37.64 ± 1.86

*AUC_*24*_ was area under the curve from 0 to 24 h; T_*max*_ was time to peak concentration. C_*max*_ was peak concentration; A was intercept for the distribution phase; B was intercept for the elimination phase; α was distribution rate constant. β was elimination rate constant; MRT was mean residence time.*

### Pharmacokinetic Characteristics of Tilmicosin in PELF

After drug administration, the tilmicosin concentration in PELF reached to a higher level at 3 h and kept at that level until 96 h. A non-compartment model was selected to analyze the drug concentration and time characteristics for PELF samples. The pharmacokinetic parameters for PELF samples are summarized in **Table 2**. The values (mean ± SD) of T_max_, C_max_, AUC_24_, and MRT were 40.80 ± 6.57 h, 5.36 ± 0.74 μg/Ml, 74.41 ± 17.98 μg.h/mL, and 37.64 ± 1.86 h, respectively.

### Comparison of Pharmacokinetics in Plasma and PELF

The concentration-time curves both in plasma and in PELF after oral administration of tilmicosin at a single dose of 40 mg/kg/bw are shown in **Figure 4**. The drug concentration in plasma and in PELF was lower than 2 μg/mL during 0~4 h. The concentration of tilmicosin in plasma rapidly decreased from 1.36 μg/mL at 6 h to 0.11 μg/mL at 72 h. In contrast, the drug concentration in PELF remained more stable, as values were higher than 4 μg/mL from 6 to 48 h and then showed a rapid decrease from 4.72 μg/mL at 48 h to 0.68 μg/mL at 96 h. Significant differences were observed between drug concentrations in plasma and in PELF. It was noteworthy that the drug concentration in PELF at 48 h reached 4.72 ± 0.44 μg/mL, which was more than 40 times of that (0.11 ± 0.03 μg/mL) detected in plasma. Both the values for C_max_ and AUC_24_ in PELF were obviously higher than those in plasma (**Table 2**).

### Monte Carlo Simulation and CO_PD_

As presented in **Table 3**, 10,000 subjects were modeled by employing Monte Carlo simulation. The PTA under different WT MICs was calculated. The PTA achieved to 100% when MIC value of 1 μg/mL was employed. However, the PTA was merely 76% (far below 90%) under the MIC value of 2 μg/mL. Consequently, the CO_PD_ was defined as 1 μg/mL.

**Table 3:** The AUC/MIC value calculated by Monte Carlo simulation and the accumulated probability of target attainment (PTA) of the target AUC/MIC (≥30) at specific MIC breakpoints.

AUC_24_ (μg.h/mL)	MIC (μg/mL)				
	0.5	1	2	4	8
47	94	47	24	12	6
94	188	94	47	24	12
69	138	69	35	17	9
76	152	76	38	19	10
63	126	63	32	16	8
87	174	87	44	22	11
…	…	…	…	…	…
PTA (%)	100	100	76	1	0

## Discussion

Temporal and geographic differences could be frequently found on the prevalence of resistance. For instance, *H. parasuis* isolates from Denmark exhibited higher MIC_90_ to tilmicosin than those isolated from Czech Republic (8 μg/mL vs. 2 μg/mL; Aarestrup et al., 2004; Nedbalcová and Kučerová, 2013). In our study, 103 *H. parasuis* isolates were collected in 2014 and antimicrobial susceptibility tests were performed using the agar dilution method according to CLSI documents. The MIC_90_ (4 μg/mL) of the 103 *H. parasuis* isolates was higher than that (2 μg/mL) of strains isolated by Zhou et al. (2010).

Wild type isolates do not harbor any acquired mutational resistance mechanisms. Hence, resistance markers specific for a microorganism-drug combination should be determined (Meletiadis et al., 2012). However, no mutations associated with tilmicosin resistance have been confirmed in *H. parasuis* isolates so far. The mutations associated with macrolide resistance in the target gene of 23S rRNA did not occur in our *H. parasuis* isolates. Conservatively, all *H. parasuis* isolates used in this study could be considered WT strains.

The bactericidal characteristic of an antibiotic is closely dependent on the organism-drug combination. The same drug is likely to show distinct bactericidal features to different bacterial strains. Most macrolide drugs exhibited time-dependent antibacterial activity and two parameters (T > MIC and AUC/MIC) were generally used for PK/PD modeling (Owens and Ambrose, 2007). However, the effect of tilmicosin against *H. parasuis* was concentration-dependent, according to the result of the *in vitro* time kill test.

In previous reports, the drug concentrations at the infection site were highly correlated to that in blood (Craig, 1998). However, as macrolide drugs may accumulate in respiratory organs, the concentrations of macrolides at the infection site were undoubtedly better to predict the clinical outcome. Although several intrapulmonary pharmacokinetic studies have been conducted using BAL in humans (Conte et al., 2000a,b, 2005; Heng et al., 2013), the BAL technique was still only limitedly applied to establish intrapulmonary pharmacokinetic models among mammal animals (Womble et al., 2007; Villarino et al., 2013). Those intrapulmonary pharmacokinetics experiments using BAL technique were normally designed for long term studies with long sampling intervals. In our study, the PK data of tilmicosin in PELF in pigs were obtained for the first time by use of the BAL technique and intrapulmonary model on the basis of our modified anesthesia method.

Although, the preparation methods for extraction of tilmicosin from plasma were available in previous studies, the extraction procedures were complex (Modric et al., 1998; Stobba-Wiley et al., 2000). To simplify the extraction procedure, we tested diverse chemical materials (acetonitrile, methyl alcohol, dichloromethane and trichloromethane) and found that dichloromethane was the optimum reagent for tilmicosin extraction. Selection of the mobile phase was important for HPLC methods. Complex chemical compositions had been required for use in the mobile phase of HPLC for detecting tilmicosin in previous studies (Shen et al., 2005; Abu-Basha et al., 2007; Herrera et al., 2007). To improve the efficiency of the HPLC method, we tried different mobile phases and found that a mobile phase with simple composition (5 mM ammonium formate: acetonitrile, 73:27, v/v) could satisfy requirements for HPLC analysis of tilmicosin in plasma and in PELF.

Drug concentrations in PELF have been positively related to antibiotic activity for treatment of intrapulmonary infections (Kiem and Schentag, 2007). Previous study found that alveolar macrophages with high concentrations of macrolide drugs could migrate into the alveolar space when organisms or dust intrude (Gladue et al., 1989). This is why we chose to establish our intrapulmonary model in healthy subjects rather than diseased animals, as the latter may influence the stability of the model. Additionally, drug concentrations in PELF in healthy animals should have more clinical relevance compared to that taken from blood in diseased animals.

Pharmacokinetics of tilmicosin in the plasma of cattle, sheep, swine and chicken have been previously described (Modric et al., 1998; Shen et al., 2005; Abu-Basha et al., 2007). The intrapulmonary pharmacokinetics of tilmicosin in swine was reported in the present study for the first time. The plasma pharmacokinetic data in this study was similar to what was reported by Shen et al. (2005). Interestingly, the concentration in PELF reached the peak value at 48 h, which was significantly later than what was detected in plasma. This may because macrolide drugs can accumulate and persist in respiratory tissues for extended periods (Villarino and Martin-Jimenez, 2013).

The CO_PD_ (1 μg/mL) was lower than the ECV (16 μg/mL) established in this study. The reasons may be: (1) the ECV was overestimated because of existing, but currently unknown, resistance mechanisms; or (2) the sample size in this study was not enough to ensure definite results. Compared to the breakpoint of tilmicosin against *A. pleuropneumoniae* and *P. multocida* (16 μg/mL), the ECV for *H. parasuis* seemed reasonable to be used as the final breakpoint of tilmicosin against *H. parasuis* in pigs.

## Author Contributions

Experiment designation: PZ, HH; Experiment carry out: PZ, JL, IA, DC, YT, ZL; Manuscript writing: PZ, HH, GC, LH, YW, MD; Manuscript review and modification: HH, ZY.

## Conflict of Interest Statement

The authors declare that the research was conducted in the absence of any commercial or financial relationships that could be construed as a potential conflict of interest.
